# Comparison of Cytological, Histopathological, and Imaging Findings Based on 10 mm Threshold in Pediatric Thyroid Nodules

**DOI:** 10.3390/children12121653

**Published:** 2025-12-05

**Authors:** Merve Cin, Burcu Özcan

**Affiliations:** Department of Pathology, University of Health Sciences, Istanbul Training and Research Hospital, Istanbul 34080, Turkey

**Keywords:** pediatric, thyroid nodules, subcentimeter, Bethesda System, USG

## Abstract

**Background/Objectives**: Both benign and malignant thyroid lesions present as nodules. While thyroid nodules are less common in the pediatric population than in adults, their malignancy rates are considerably higher. Although the 10 mm cut-off for fine-needle aspiration cytology (FNAC) is commonly used for both adults and children, there is limited information regarding subcentimeter thyroid nodules in the pediatric population. The majority of published studies have focused on nodules measuring 1 cm or greater. This study aimed to compare the cytological diagnosis, ultrasonographic features, and histopathological outcomes of thyroid nodules in pediatric patients (under 21 years old), stratified by size (≤10 mm vs. >10 mm). **Methods**: We conducted a retrospective, single-center cohort study, evaluating 108 thyroid nodules from 98 patients. Nodule sizes were categorized into two groups, and their features were correlated with findings from FNAC using the Bethesda System for Reporting Thyroid Cytopathology and subsequent surgical histopathology. The risk of malignancy (ROM) was calculated for each Bethesda category. **Results**: A total of 108 nodules were evaluated, with 35 (32.4%) measuring ≤ 10 mm. The overall malignancy rate was 12%, with 14.3% in the ≤10 mm group and 11% in the >10 mm group. The difference was not statistically significant, and this finding indicates that small nodules can also harbor malignancy. Notably, all cases categorized as suspicious for malignancy or malignant by FNAC were confirmed to be malignant on histopathology (ROM = 100%). The Atypia of Undetermined Significance (AUS) category exhibited a malignancy rate of 60%, which is significantly higher than the rates reported in previous studies. Ultrasonographic features such as hypoechogenicity and microcalcifications were more prevalent in malignant nodules but lacked statistical significance. **Conclusions**: Our findings demonstrate that pediatric thyroid nodules, including those ≤10 mm, have a notable risk of malignancy. The high rate of malignancy in the AUS category suggests that the current Bethesda criteria, primarily designed for adults, may require re-evaluation for pediatric cases due to known differences in genetic profiles and disease behavior. Consequently, these pathological findings clearly demonstrate that FNAC indications in children should not be based solely on nodule size, and that a multidisciplinary approach guided by pediatric-specific guidelines should inform clinical management.

## 1. Introduction

Although thyroid nodules are less common in children than in adults, the risk of malignancy is significantly higher in pediatric patients [1]. The reported incidence of thyroid nodules in adults ranges from 4% to 50%, while in children, it is about 1% to 2% [2,3,4]. However, the malignancy rate of thyroid nodules in children can reach as high as 19–29.2%, which is higher than the 5–12% observed in adults [1,4,5]. According to SEER data, thyroid cancers in individuals under the age of 20 make up roughly 1.8% of all cases in the United States [6]. Although the overall incidence has been rising recently, thyroid cancer is diagnosed five times more often in adolescent girls aged 15–19 than in boys of the same age group, and it usually presents as a palpable nodule [6]. These findings highlight the need for a distinct approach to diagnosing and managing thyroid nodules in pediatric patients that differs from protocols used for adults.

Fine-needle aspiration cytology (FNAC) is a standard diagnostic method used to assess the risk of malignancy in both pediatric and adult thyroid nodules [7]. According to the Bethesda System (BS) for Reporting Thyroid Cytopathology, which is widely used worldwide, pediatric patients show a significantly higher risk of malignancy in nodules classified as atypia of undetermined significance (AUS) and follicular neoplasm (FN). The average malignancy rates for these categories are approximately 28% and 50% in children, respectively, compared to 22% and 30% in adults [8]. In pediatric groups, malignancy rates exceeding 50% in the AUS category and nearly 100% in the FN category have been reported [8]. Additionally, even in nodules labeled as benign on cytology, a malignancy risk from 0.7% to 27% has been observed in children [1,4,8].

There are also significant differences in molecular profiling between pediatric and adult cases. In pediatric thyroid carcinomas, RET/PTC fusions are more commonly observed (ranging from 37% to 87%), while BRAF and RAS mutations are rarely detected (0–16% and 0–6%, respectively) [6,9,10,11]. Due to the lack of validated diagnostic molecular tests in pediatric patients, surgical intervention is often prioritized—especially in indeterminate cytological categories—to prevent the risk of delayed cancer diagnosis [6,11].

Ultrasonography (USG) is central to detecting and characterizing thyroid nodules [12]. While the American Thyroid Association (ATA) guidelines recommend FNAC evaluation for nodules larger than 10 mm in adults, primary thyroid malignancies with metastases have been reported in nodules smaller than 10 mm in pediatric populations [6]. Furthermore, the surgical management of subcentimeter (<10 mm) nodules diagnosed as papillary thyroid carcinoma (PTC) via FNAC differs between adults and children. In adults, lobectomy is usually sufficient if there is no extrathyroidal extension or regional lymph node metastasis. However, in pediatric patients, total thyroidectomy is recommended regardless of nodule size [13]. Nonetheless, considering the risk of surgical complications like recurrent laryngeal nerve injury and hypoparathyroidism, clinical follow-up or repeat FNAC remains a viable alternative in low-risk cytological categories, such as AUS [7].

Numerous studies in the literature have assessed FNAC outcomes and malignancy risk in pediatric thyroid nodules [1,2,4,5,14,15,16]. A large multicenter cohort study by Canberk et al. [16] examined the performance of the Bethesda classification system in pediatric thyroid nodules in detail, highlighting the notably high malignancy rates. While the study noted that 13% of the nodules were smaller than 10 mm, specific data on the malignancy rates of these micronodules were not thoroughly analyzed [16]. Although many studies link increased nodule size with a higher malignancy risk, there is an emphasis on the inconsistent correlation between nodule size and malignancy, underscoring the importance of clinical and radiological features [17,18]. Notably, there is a lack of studies focusing specifically on nodules smaller than 10 mm (i.e., micronodules or microcarcinomas) in young patients, with limited data analyzing malignancy rates within this subgroup in detail.

In this context, our study aimed to investigate the relationship between nodule size (≤10 mm vs. >10 mm) and cytological classification, histopathological outcomes following surgical resection, and radiological characteristics in individuals under 21 years of age, thereby providing a pathological perspective that may inform risk-oriented management strategies independent of nodule size.

## 2. Materials and Methods

This retrospective cohort study was conducted at a single center (Istanbul Training and Research Hospital, Department of Pathology) from January 2018 to May 2025 to compare thyroid nodules smaller than 10 mm with those larger than 10 mm in individuals younger than 21 years of age. The upper age limit was established as 21 years, in accordance with the recommendations of the American Academy of Pediatrics [19]. A total of 98 patients with thyroid nodules measured by USG, involving 108 nodules, were included in the study. In cases where multiple fine-needle aspiration (FNA) procedures were performed on the same nodule at different times, only the most recent cytology specimen was analyzed (n = 6). For patients with multiple nodules, each nodule was evaluated independently regarding its Bethesda category (and histopathology if present). Specifically, the cohort included 18 nodules from 8 patients (two patients presented with three nodules each, and six with two).

Based on data collected retrospectively from the hospital’s electronic medical record system, nodule size in USG evaluations was measured in two axes (longitudinal and transverse), with the largest diameter used as the reference. Nodules were classified into two groups: ≤10 mm and >10 mm. Additionally, ultrasonographic features such as echogenicity (isoechoic, hypoechoic, hyperechoic, and heterogeneous) and the presence or absence of microcalcifications were recorded. Only the ultrasonographic information provided by radiology (nodule size, echogenicity, microcalcifications) and recorded on the pathology request forms was available for analysis. Other clinical data, including history of radiation exposure, autoimmune thyroiditis, or genetic syndromes, were not included.

All nodules that underwent FNAC were classified I–VI according to the 2023 Bethesda System for Reporting Thyroid Cytopathology [8]: Non-diagnostic (ND): I, Benign: II, Atypia of Undetermined Significance (AUS): III, Follicular Neoplasm (FN): IV, Suspicious for Malignancy (SFM): V, and Malignant: VI. Surgical resection was performed on 23 nodules from 19 patients. Cytological and histopathological evaluations were conducted in a blinded manner by two experienced pathologists, one of whom specializes in cytopathology. Histopathological diagnoses were based on the WHO Classification of Endocrine and Neuroendocrine Tumours (5th edition) [20]. Non-invasive follicular thyroid neoplasm with papillary-like nuclear features (NIFTP) was classified as benign according to the WHO 5th edition guidelines. Demographic data (age, sex) were obtained from the pathology report archives.

The risk of malignancy (ROM) for nodules classified according to the BS was calculated using two methods. The histopathology-based malignancy rate, referred to as ROM-H, was defined as the number of malignant nodules confirmed by histopathology divided by the total number of resected nodules within that cytological category (e.g., number of malignant nodules in the AUS group/total number of resected AUS nodules). Similarly, ROM-C was calculated by dividing the number of nodules confirmed as malignant after resection by the total number of nodules assigned to each cytological category, regardless of whether surgery was performed (e.g., number of malignant resected nodules in the AUS group/total number of nodules categorized as AUS by FNAC). As expected, ROM-H yielded a higher rate, since nodules with benign or indeterminate features are less likely to undergo surgical resection.

For descriptive statistics, continuous nonparametric variables were expressed as median (minimum-maximum), and categorical variables were expressed as percentages. Variables that were not normally distributed between the two groups were analyzed using the Mann–Whitney U test. The Chi-square test or Fisher’s Exact test was used to compare categorical variables. A *p*-value of <0.05 was considered statistically significant in all analyses.

This study was approved by the Istanbul Training and Research Hospital Clinical Research Ethics Committee (Number: 161, Date: 27 June 2025) and was conducted in accordance with the principles of the Helsinki Declaration.

## 3. Results

A total of 108 thyroid nodules from 98 patients were evaluated (Table 1). Of these patients, 79 (80.7%) were female and 19 (19.3%) were male. All patients with multiple nodules (n = 8) were female. Patient ages ranged from 8 to 20 years, with a mean of 17.66 ± 2.8 years. Regarding localization, 60 nodules (55.5%) were found in the right lobe, 41 (37.9%) in the left lobe, and 7 (6.6%) in the isthmus. Nodule sizes ranged from 5 to 50 mm, with a mean diameter of 17.3 mm.

Based on cytological findings, 14 nodules (13%) were classified as ND, 63 (58.3%) as benign, 20 (18.5%) as AUS, 5 (4.6%) as SFM, and 6 (5.6%) as malignant (Table 2). No cases were diagnosed as FN. Surgical resection was performed in 23 nodules (21.2%). Histopathological examination showed that 10 nodules (9.2%) were benign, including five cases of adenomatoid hyperplasia, three follicular adenomas, one lymphocytic thyroiditis, and one NIFTP. Thirteen nodules (corresponding to 12% of the total cohort) were malignant: 10 of these (77% of malignant cases) were classic papillary thyroid carcinoma (PTC), while one case each (7.6%) was diagnosed as the follicular variant of PTC, medullary carcinoma, and poorly differentiated thyroid carcinoma (Table 3). No nodules from the ND group underwent surgical resection.

Radiological data were available for echogenicity in 103 nodules and for the presence of microcalcifications in 99 nodules. The majority of nodules (n = 83, 80.6%) were hypoechoic, while only one nodule (1%) was hyperechoic. Microcalcifications were detected in 17 (17.2%) of the nodules (Table 1).

The risk of malignancy (ROM) was calculated for all nodules based on their Bethesda category, using both cytologic (ROM-C) and histopathologic confirmation (ROM-H). ROM could not be determined for the ND category, as no nodules in this group underwent surgical resection. Among the 63 nodules in the benign category, 8 (12.7%) were resected, and none were found to be malignant (ROM-C and ROM-H = 0%). Of the 20 AUS nodules, 5 (25%) were resected, with malignancy confirmed in 3 cases (ROM-C = 15%, ROM-H = 60%). All five nodules in the SFM category were resected and confirmed as malignant (ROM-C and ROM-H = 100%). In the malignant category, six nodules were identified; 5 of these were resected, and all were confirmed malignant, while histopathological follow-up data were unavailable for one case (ROM-C = 83.3%, ROM-H = 100%). Additionally, ROM-C and ROM-H values for nodules ≤ 10 mm and >10 mm are presented in Table 4.

## 4. Discussion

The present study, we compared the cytological, histological, and ultrasonographic characteristics of thyroid nodules in a pediatric population younger than 21 years, stratified by size (≤10 mm versus >10 mm). Our results showed that the ≤10 mm group had a similar malignancy rate (14.3%) to the >10 mm group (11%). Although this difference was not statistically significant, it shows that malignancy may also occur in small thyroid nodules in children [18,21,22,23,24].

FNAC is generally recommended for patients with palpable nodules, nodules larger than 10 mm, or in the presence of additional risk factors such as radiation exposure or suspicious ultrasonographic features [21]. In our study, we chose the 10 mm threshold because it is the widely accepted size cutoff for FNAC indication. According to the BS, all nodules in both the ≤10 mm and >10 mm groups that were categorized as SFM or malignant (categories V and VI) were confirmed to be malignant on histological follow-up (n = 10, ROM-H = 100%). Although rare, current guidelines indicate that the likelihood of malignancy in pediatric thyroid nodules is higher than in adults, and they recommend that the decision for cytopathological evaluation be based on clinical and radiological findings regardless of nodule size [23,24].

The majority of studies [4,5,15,25] in the literature do not report detailed data regarding nodule size, while Cherella et al. [1] included only nodules larger than 1 cm in their study. Canberk et al. [16] reported that 13% (n = 22) of nodules in their series measured less than 10 mm. In our cohort, nodules measuring ≤ 10 mm made up 32.4% (n = 35) of the total, with 18 nodules (16.6%) measuring less than 10 mm. The comparatively higher proportion of ≤10 mm nodules in our study may reflect institution-specific or regional differences in referral patterns.

The reported rate of ND cytology in the literature ranges from 8% to 26% [1,5,7,16,25]. In our study, the ND rate was 22.9% for nodules measuring ≤ 10 mm and 8.2% for those >10 mm, with an overall ND rate of 13%. Although our findings fall within the reported range, it is clear that ND samples are significantly more common in subcentimeter nodules.

Benign cytology accounted for 58.3% of all FNACs (48.6% in the ≤10 mm group and 63% in the >10 mm group), which aligns with reported benign rates of 50–71% in the literature [1,5,7,16,25]. In a study focusing exclusively on nodules ≥ 10 mm, Cherella et al. [1] reported a benign rate of 64%. Our findings support those of McHenry et al. [18], demonstrating a tendency for larger nodules to be benign and reinforcing the idea that nodule size alone should not be overemphasized in malignancy risk stratification.

In the 2023 edition of the BS, the term “follicular lesion of undetermined significance” was removed, and the category was simplified to “atypia of undetermined significance” (AUS) [8]. While the frequency of AUS is reported to be similar between adult and pediatric populations, the ROM in pediatric cases is approximately twice as high as in adults [1,8]. In the literature, the prevalence of AUS in children ranges from 7% to 11.3%, with ROM-C values reported between 10% and 44%, and ROM-H between 20% and 54% [1,5,7,16]. In study, both the AUS rate (18.5%) and the ROM-H value for the AUS group (60%) were higher than those reported in previous series (Table 5). The rate of AUS was 14.3% among nodules ≤ 10 mm and even higher among nodules > 10 mm (20.6%). Additionally, the proportion of cytologically malignant cases in our study (5.6%) was lower than the rates reported in the literature (7–14%) [1,5,7,16]. This discrepancy may be attributable to regional variation, interobserver variability, or differences in study design; however, it concurrently raises the possibility that some malignant cases were classified as AUS. Cherella et al. [1] suggested that autoimmune thyroiditis—which is more common in adults—may mimic cytological atypia, proposing this as a potential explanation for the higher malignancy rates seen in indeterminate categories (AUS or FN) in the pediatric population. Still, in the same study, the AUS rate was reported as 7% for both adults and children, indicating that AUS is not more common in adults. We do not believe, however, that this hypothesis fully explains the high ROM in the AUS group among pediatric patients. We hypothesize that, in children, cytopathologists or pathologists may tend to avoid assigning definitive, alarming diagnoses (SFM or malignant) due to concerns about surgical complications and lifelong hormone replacement therapy. Nevertheless, this remains a speculative observation, as no specific measurement or evaluation to confirm this has been performed in this study.

Although PTC is the most common thyroid malignancy in both pediatric and adult populations, established molecular differences—such as the predominance of BRAFV600E and RAS mutations in adults versus RET, NTRK, ALK, and MET alterations in children—may result in cytomorphological differences [11]. Moreover, in children, the malignancy rate in the FN category can reach up to 100%, compared to approximately 30% in adults [8]. Given these distinct genetic alterations and higher malignancy rates, we propose that the Bethesda diagnostic criteria for AUS and FN—originally designed based on adult populations—should be reconsidered and potentially revised for pediatric patients to include age-specific features. In line with this, Tuli et al. [26] reported that the Italian classification (SIAPEC 2014) demonstrated higher sensitivity than the BS in differentiating benign from malignant lesions within these indeterminate categories.

The 2015 ATA guidelines recommended surgery for nodules classified as AUS, while the 2023 edition of the BS added repeat FNAC as an alternative approach [6,8]. In a 2019 series, 72% of cases initially diagnosed as AUS were reclassified after repeat FNAC, with 17% shifting into the SFM or malignant categories [1]. Some studies have grouped AUS, FN, and SFM under a single “indeterminate” category [5,6,15]. In our study, the malignancy rate in the AUS group was 60%, and—as reported in other series—the SFM group showed a malignancy rate of 100% [5,7]. Given this significant difference in malignancy risk, we conclude that combining AUS and SFM into a single indeterminate category does not appear appropriate, particularly in the pediatric setting.

In our series, 12% of nodules (n = 13) were diagnosed as malignant based on histopathological examination. Among these, three were initially classified as AUS, while five nodules each were categorized as SFM and malignant, respectively. Out of the histologically confirmed malignant cases, eight were larger than 10 mm. In other words, 38.4% of the resected malignant nodules measured ≤ 10 mm. All these ≤10 mm malignant cases showed typical PTC morphology. As reported in the literature, the most common histological subtype in our study was PTC, found in 84.6% of malignant cases (11 out of 13), of which 91% (n = 10) were of the classic variant [1,5,7,15,16]. From a pathological perspective, subcentimeter nodules may show malignancy rates at least as high as those of larger nodules, suggesting that size alone is not a reliable criterion for risk stratification. Moreover, the high malignancy rates identified in cytological categories such as AUS and SFM in our study may justify earlier surgical evaluation. Furthermore, given the challenges linked to indeterminate cytology, incorporating molecular or genetic testing could further improve clinical management approaches.

In many studies, the SFM and malignant categories in pediatric populations are linked to very high ROM values, ranging from 65% to 100% [1,5,7,15,16]. Although slight variability was observed in ROM-C and ROM-H values, both were found to be 100% in the SFM group in our study, while the malignant group showed a ROM-C of 83.3% and a ROM-H of 100%. These findings, consistent with the literature, indicate that the BS offers reliable and consistent diagnostic guidance for clinicians in patient management, especially within the SFM and malignant categories.

In some series, discordance between cytological and histopathological outcomes has been reported in nodules classified as either benign or malignant by FNAC [5,16,25]. Among cases with benign cytology, ROM values have been reported to range from 0.7% to 15% [1,5,16]. In our study, no malignancy was found in any of the resected nodules classified as benign in cytology (ROM = 0%). A notable finding in our cohort was that the resection rates for ND, benign, and AUS categories were relatively low (0%, 12%, and 25%, respectively) compared to those reported in the literature. Previous studies have documented resection rates as high as 38% for ND, 28% for benign, and up to 81% for AUS nodules [1,5,16]. These results suggest that, at our institution, surgical resection is generally reserved for cases that carry concerning cytological diagnoses (such as SFM or malignant) or suspicious radiological features. This indicates a more conservative approach in clinical management and suggests partial non-adherence to the 2015 ATA guideline recommendations. This approach likely also explains the high malignancy rate (ROM-H = 60%) in the few nodules resected in our AUS group.

In USG evaluation, several suspicious features have been described. These include solid and hypoechoic composition, the presence of microcalcifications, a taller-than-wide shape, irregular margins, and evidence of extrathyroidal extension. Such findings are categorized as “highly suspicious” [12]. In our study, 85.7% of nodules in the ≤10 mm group were hypoechoic, compared to 72.6% in the >10 mm group. Among the histologically confirmed malignant nodules, 84.6% were hypoechoic, and 23% exhibited microcalcifications. In contrast, among the benign nodules based on FNAC, 73% were hypoechoic, and 12.6% showed microcalcifications. Jang et al. [27] reported that 72.7% of benign nodules were hypoechoic, and 10% had calcifications, whereas 99% of malignant nodules were hypoechoic, and 20.8% had calcifications, but these differences were not statistically significant (hypoechogenicity, *p* = 0.064; calcification, *p* = 0.098). Although hypoechogenicity and microcalcifications appeared more often in malignant nodules, their correlation with malignancy lacked statistical significance. As noted by Martinez-Rios et al. [12], individual USG findings have limited diagnostic accuracy in differentiating benign from malignant nodules. Most previous studies in the literature have focused on nodules larger than 1 cm; to our knowledge, this is the only study that evaluates USG features and ROM rates in small (≤10 mm) pediatric thyroid nodules using a Bethesda-based classification.

This study has several limitations, such as its retrospective, single-center design, limited sample size, and lack of histopathological confirmation for all cases. Additionally, molecular profiling data were not available. Another important limitation is the absence of clinical information for the patients (such as data on genetic syndromes, prior radiation exposure, or autoimmune thyroiditis).

## 5. Conclusions

We evaluated thyroid nodules measuring ≤10 mm and >10 mm in patients under 21 years old, focusing on cytological diagnosis, ultrasonographic features, and histopathological outcomes. Among the small nodules (≤10 mm), we found a malignancy rate of 14.3%. Additionally, 60% of the resected nodules initially diagnosed as AUS were confirmed to be malignant. Considering the known differences in genetic alterations between adult and pediatric thyroid carcinomas, and the high ROM reported for the AUS category, we suggest reassessing the cytomorphological criteria within the BS for pediatric populations. This study has been supported with pathological data that FNAC indications should not be based solely on nodule size. Moreover, clinical management could be improved through a multidisciplinary tumor board approach that includes detailed clinical history, imaging findings, and, especially for indeterminate nodules, preoperative molecular analyses with proven diagnostic utility.

## Figures and Tables

**Table 1:** Comparison of demographic, radiological, and histological characteristics of the ≤10 mm and >10 mm groups.

Variable	≤10 mm (n = 35)	>10 mm (n = 73)	*p* Value
Age, mean [min–max, years]	17, 6 [8–20]	17, 6 [8–20]	0.954
Sex			0.320
Female, n (%)	27 (77.1)	62 (84.9)	
Male, n (%)	8 (22.9)	11 (15.1)	
Localization			0.079
Right lobe, n (%)	19 (54.3)	41 (56.2)	
Left lobe, n (%)	11 (31.4)	30 (41.1)	
Isthmus, n (%)	5 (14.3)	2 (2.7)	
USG echogenicity			0.644
Isoechoic, n (%)	3 (8.6)	7 (9.6)	
Hypoechoic, n (%)	30 (85.7)	53 (72.6)	
Hyperechoic, n (%)	0 (0.0)	1 (1.4)	
Heterogeneous, n (%)	2 (5.7)	7 (9.6)	
Not available, n (%)	-	5 (6.8)	
Microcalcification			0.514
Present, n (%)	7 (20.0)	10 (13.7)	
Absent, n (%)	27 (77.1)	55 (75.3)	
Not available, n (%)	1 (2.9)	8 (11)	
Histological malignancy, n (%)	5 (14.3)	8 (11)	0.179

**Table 2:** Distribution of ≤10 mm and >10 mm nodules according to the Bethesda system.

	I-ND n = 14	II-Benign n = 63	III-AUS n = 20	V-SFM n = 5	VI-Malignant n = 6	*p* Value
**≤10 mm** n = 35 (%)	8 (22.9)	17 (48.6)	5 (14.3)	3 (8.5)	2 (5.7)	0.158
**>10 mm** n = 73 (%)	6 (8.2)	46 (63)	15 (20.6)	2 (2.7)	4 (5.5)	

ND: Non-diagnostic, AUS: Atypia of undetermined significance, SFM: suspicious for malignancy.

**Table 3:** Histopathological follow-up results based on nodule size (≤10 mm and >10 mm) and Bethesda categories.

	Adenomatous Hyperplasia	Lymphocytic Thyroiditis	Follicular Adenoma	NIFTP	Classic PTC	FV-PTC	Medullary Carcinoma	Poorly Differentiated Thyroid Carcinoma
**≤10 mm** n = 6	1	0	0	0	5	0	0	0
**>10 mm** n = 17	4	1	3	1	5	1	1	1
Bethesda category								
**ND**	-	-	-	-	-	-	-	-
**Benign**	5	1	2	-	-	-	-	-
**AUS**	-	-	1	1	1	1	-	1
**SFM**	-	-	-	-	5	-	-	-
**Malignant**	-	-	-	-	4	-	1	-

ND: Non-diagnostic, AUS: Atypia of undetermined significance, SFM: suspicious for malignancy, NIFTP: Non-invasive follicular thyroid neoplasm with papillary-like nuclear features, PTC: Papillary thyroid carcinoma, FV-PTC: Follicular variant papillary thyroid carcinoma.

**Table 4:** Risk of malignancy rates (ROM-C, ROM-H) of ≤10 mm and >10 mm nodules based on cytology and histology.

	Bethesda Category	Number of FNACs (n = 108)	Number of Histological Follow-Ups (n = 23)	ROM-C (%)	ROM-H (%)
**≤10 mm**	ND (I)	8	0	NA	NA
	Benign (II)	17	1	0	0
	AUS (III)	5	0	NA	NA
	SFM (V)	3	3	100	100
	Malignant (VI)	2	2	100	100
Total		35	6		
**>10 mm**	ND (I)	6	0	NA	NA
	Benign (II)	46	7	0	0
	AUS (III)	15	5	20	60
	SFM (V)	2	2	100	100
	Malignant (VI)	4	3	75	100
Total		73	17		

NA: Not applicable, ND: Non-diagnostic, AUS: Atypia of undetermined significance, SFM: suspicious for malignancy, FNAC: Fine-needle aspiration cytology, ROM-C: Risk of malignancy-cytology, ROM-H: Risk of malignancy-histopatology.

**Table 5:** Malignancy Rates (ROM) of Pediatric Thyroid Nodules According to Bethesda Categories: A Comparison of Our Series’ Data with Selected Studies.

Reference	Population	Nodule Size (mm)	Year	Total FNAC No	ND (ROM-C–ROM-H)	Benign (ROM-C–ROM-H)	AUS (ROM-C–ROM-H)	FN (ROM-C–ROM-H)	SFM (ROM-C–ROM-H)	M (ROM-C–ROM-H)
Amirazodi et al. [15]	<18 years, Canada	-	2016	207	0%	3–16%	35–67% *		50–71%	95–100%
Cherella et al. [1]	<19 years, USA	≥10	2019	430	11–30%	0.7–3%	44–54%	71%	73–76%	97–100%
Wang et al. [5]	≤21 years, USA	-	2019	302	0%	1.8–7%	11.5–20%	20–25%	100%	85.7–100%
Jiang et al. [4]	≤18 years, USA	-	2021	203	13.8–33.3%	4.7–26.3%	22.7–31.5%	35.7–38.4%	83.3%	100%
Canberk et al. [16]	<21 years, Portugal-Turkey	- **	2022	405	7–30%	2.5–15%	15–33%	39–52%	79–86%	67–88%
Current study	<21 years, Turkey	≤10	2025	35	0%	0%	0%	-	100%	100%
		>10		73	0%	0%	20–60%	-	100%	83.3–100%

FNAC: Fine-needle aspiration cytology, ND: Non-diagnostic, AUS: Atypia of undetermined significance, FN: Follicular neoplasm, SFM: suspicious for malignancy, M: Malignant. * Amirazodi et al. combined categories III and IV under the heading of “atypical.” These ROM values are for the atypical group. ** Canberk et al. reported that 13% of the nodules were smaller than 10 mm.

## Data Availability

The data presented in this study are available on request from the corresponding author due to privacy and ethical reasons.
